# Clinical Outcomes According to Age and Atrial Fibrillation Type: Insights from the Multicentre REGUEIFA Registry

**DOI:** 10.3390/jcm15187295

**Published:** 2026-09-19

**Authors:** Alejandro Manuel López-Pena, Juliana Elices-Teja, Olga Durán-Bobín, Laila González-Melchor, María Vázquez-Caamaño, Emiliano Fernández-Obanza, Eva González-Babarro, Pilar Cabanas-Grandío, Miriam Piñeiro-Portela, Oscar Prada-Delgado, Mario Gutiérrez-Feijoo, Evaristo Freire, Oscar Díaz-Castro, Javier Muñiz, Eduardo Barge-Caballero, Javier García-Seara, Carlos González-Juanatey

**Affiliations:** 1 Cardiology Department, Hospital Universitario Lucus Augusti, 27003 Lugo, Spain; alejandro.manuel.lopez.pena@sergas.es (A.M.L.-P.); juliana.elices.teja@sergas.es (J.E.-T.); olga.duran.bobin@sergas.es (O.D.-B.); laila.gonzalez.melchor@sergas.es (L.G.-M.); 2 CardioHULA Research Group, Fundación Instituto de Investigación Sanitaria de Santiago de Compostela FIDIS, 27003 Lugo, Spain; 3 Cardiology Department, Hospital San Rafael, 15006 La Coruña, Spain; macrisvc@yahoo.es; 4 Cardiology Department, Hospital Álvaro Cunqueiro and Instituto de Investigación Sanitaria Galicia Sur (IISGS), 36312 Vigo, Spain; emilio.fernandez-obanza-windcheid@sergas.es (E.F.-O.); pilar.cabanas.grandio@sergas.es (P.C.-G.);; 5 Cardiology Department, Hospital Montecelo, 36071 Pontevedra, Spain; eva.gonzalez.babarro@sergas.es; 6 Cardiology Department, Complexo Hospitalario Universitario de A Coruña, Instituto de Investigación Biomédica de A Coruña (INIBIC), 15006 A Coruña, Spain; miriam.pineiro.portela@sergas.es (M.P.-P.); oscar.prada.delgado@sergas.es (O.P.-D.); eduardo.barge.caballero@sergas.es (E.B.-C.); 7 Cardiology Department, Hospital de Ourense, 32005 Ourense, Spain; mario.gutierrez.feijoo@sergas.es (M.G.-F.); evaristo.freire@sergas.es (E.F.); 8 Grupo de Investigación Cardiovascular, Departamento de Ciencias de la Salud and Instituto de Investigación Biomédica de A Coruña (INIBIC), Universidade da Coruña, CIBERCV, 15006 A Coruña, Spain; javier.muniz.garcia@udc.es; 9 Cardiology Department, Hospital Clínico de Santiago de Compostela, CIBERCV, 15706 A Coruña, Spain; javiergarciaseara@yahoo.es; 10 Nursing School of Santiago de Compostela University (Campus Lugo), 2001 Lugo, Spain

**Keywords:** atrial fibrillation, age, prognosis, heart failure, stroke, mortality, atrial fibrillation type, real-world study, comorbidity

## Abstract

**Background/Objectives**: Atrial fibrillation (AF) represents the most common sustained cardiac arrhythmia, and its incidence rises markedly with advancing age. The relative prognostic impact of age compared with AF type remains uncertain. This study assessed the association between age, AF type, and clinical outcomes in a large real-world AF cohort. **Methods:** This study was based on data from the prospective, observational, multicentre REGUEIFA registry, which includes consecutive patients with AF treated at eight hospitals in northwestern Spain. Patients were stratified by age and AF type. Baseline characteristics, comorbidities, treatments, and risk scores were collected. Patients were followed for at least 2 years, and outcomes included all-cause mortality, heart failure (HF) worsening, stroke, and a composite endpoint. **Results:** A total of 997 patients were included. Increasing age was associated with a worse clinical profile, with higher comorbidity burden and increased thromboembolic and bleeding risk. During follow-up, adverse events increased stepwise with age, including mortality, HF worsening, and the composite endpoint. AF type was not independently associated with outcomes after multivariable adjustment. Rhythm-control strategy was independently associated with a lower risk of the composite endpoint. Age remained an independent predictor of all outcomes. Risk of the composite endpoint increased across age groups (HR 2.01 for 75–84 years; 2.34 for 85–89 years; 6.50 for ≥90 years vs. <75 years). Each additional year of age was associated with a 9% higher mortality risk (HR 1.09; 95% CI 1.06–1.13). **Conclusions:** In this prospective real-world cohort of patients with AF, age was the strongest and most consistent predictor of adverse clinical outcomes, whereas AF type lost prognostic significance after adjustment. These findings support age and overall clinical burden as key determinants of prognosis, favouring a patient-centred approach over AF subtype classification.

## 1. Introduction

Atrial fibrillation (AF) represents the most common sustained arrhythmia and is linked to a greater risk of adverse clinical outcomes, including heart failure (HF), ischaemic stroke, and premature mortality [1,2].

Age is a major determinant of AF development, and its incidence doubles with every additional decade of life [3]. In our setting, the prevalence of AF is estimated to be approximately 4.4% among adults aged over 40 years, increasing to as high as 17% in individuals older than 80 years [4]. The burden of AF is expected to rise substantially over the coming decades; by 2050, its prevalence is projected to reach 2.5 times its current level, and over half of affected individuals are expected to be aged 80 years or older [5,6].

According to its temporal pattern, AF is classified as paroxysmal, persistent, permanent, or first-diagnosed [1]. The prognostic significance of AF type, particularly with regard to stroke risk, remains controversial, with previous studies reporting conflicting results [7,8,9]. Current clinical guidelines recommend that the indication for oral anticoagulation should be based on an individual assessment of thromboembolic risk using established risk scores, including the CHA_2_DS_2_-VA score, rather than on the temporal pattern of the arrhythmia [1,7]. Nevertheless, the independent prognostic impact of AF type on clinical outcomes, including stroke, HF, and mortality, remains a matter of ongoing debate.

Older patients with AF have a higher thromboembolic risk profile as well as an increased risk of bleeding [1,10,11]. They also tend to have a higher comorbidity burden and are more frequently affected by polypharmacy and frailty than younger individuals [10,11,12,13]. Furthermore, patients with non-paroxysmal AF tend to be older and have a greater burden of cardiovascular risk factors and comorbidities than those with paroxysmal AF [14,15].

AF confers a higher risk of adverse cardiovascular outcomes during follow-up [1,2]. Previous studies have demonstrated a bidirectional relationship between AF and HF, whereby each condition may precipitate the onset or progression of the other, and both frequently coexist in clinical practice [16]. In this context, HF is a major contributor to cardiovascular mortality among patients with AF. In the Framingham Heart Study, the onset of HF among patients with AF was associated with an approximately three-fold higher risk of death [17]. In addition, AF-related cardioembolism is responsible for up to one-third of ischaemic strokes, which are more likely to result in death or long-term disability than strokes of other aetiologies [18].

This study aimed to assess the incidence of all-cause mortality, HF, and ischaemic stroke over the follow-up period in a large prospective multicentre cohort of patients with AF enrolled in the Registro Gallego Intercéntrico de Fibrilación Auricular (REGUEIFA), and to investigate the association of age and AF type with these clinical outcomes [19].

## 2. Material and Methods

### 2.1. Study Design

The present study was based on data from the prospective, observational, multicentre REGUEIFA registry, which includes patients with AF treated at eight hospitals within a single healthcare area in northwestern Spain (Galicia). The overall study design and the characteristics of the registry have been described previously [19].

In brief, patients with AF, either as a primary or secondary diagnosis, were consecutively enrolled following evaluation at outpatient cardiology clinics or admission to cardiology departments at the participating centres from January 2018 to February 2020.

### 2.2. Selection of Participants

Eligible participants were adults (≥18 years) of either sex with a confirmed diagnosis of AF. The diagnosis of AF was established using a standard 12-lead electrocardiogram or ambulatory electrocardiographic monitoring, including external Holter monitoring or implantable cardiac devices, documenting AF episodes lasting more than 30 s. Eligibility also required at least one documented episode of AF during the year before enrolment. Patients could be in sinus rhythm at the baseline visit provided they fulfilled the diagnostic criteria described above.

Patients for whom long-term clinical follow-up was not anticipated were excluded, together with those presenting with secondary or transient AF related to reversible causes. Patients participating in interventional clinical trials that could substantially influence treatment, follow-up frequency, or diagnostic strategies were also excluded.

### 2.3. Ethical Statement

Before enrolment, participants received detailed information about the study and provided written informed consent. The study adhered to the principles of the Declaration of Helsinki and was approved by the Galician Clinical Research Ethics Committee (Comité de Ética de la Investigación Clínica de Galicia, Spain) (reference number 2016/376; approval date: 15 November 2016).

### 2.4. Data Registry and Quality Assurance

Clinical information was recorded using a dedicated electronic case report form (eCRF) specifically developed for the registry. Data were entered by investigators at each participating centre and transmitted to the coordinating centre through a secure web-based platform, ensuring data confidentiality and compliance with the General Data Protection Regulation (GDPR) and other applicable data protection regulations. Each participant was assigned a unique numerical identification code to guarantee data anonymisation.

To ensure data quality, a monitoring process was implemented in which an independent study monitor reviewed a random sample of 10% of the electronic case report forms across participating centres by comparing the recorded information with the original source documentation.

### 2.5. Methods and Measurements

For this analysis, patients were stratified into four age groups (<75, 75–84, 85–89, and ≥90 years) to capture clinically relevant differences, with age also analysed as a continuous variable in the multivariable analysis. They were also classified by AF type as first-diagnosed AF, paroxysmal AF, persistent AF, and long-standing persistent/permanent AF. First-diagnosed AF was defined as newly diagnosed AF, regardless of symptom status, temporal pattern, or duration. Paroxysmal AF was defined as AF terminating within 7 days, whereas persistent AF was defined as AF lasting >7 days or requiring intervention for termination. Long-standing persistent AF was defined as continuous AF lasting ≥12 months when rhythm control remained a treatment option, and permanent AF as AF for which no further rhythm restoration was planned [1].

At baseline, demographic and clinical characteristics were recorded, including age, sex, cardiovascular risk factors, previous cardiovascular disease, and relevant comorbidities. Available complementary investigations, including laboratory and echocardiographic parameters, as well as clinical characteristics of AF, were also recorded.

Current pharmacological treatment was documented, including antiarrhythmic therapy, cardiovascular medications, and oral anticoagulant therapy. Thromboembolic and bleeding risks were evaluated using the CHA_2_DS_2_-VASc and HAS-BLED scores, respectively.

All patients completed a minimum follow-up of 2 years. Follow-up data included all-cause mortality, heart failure (new-onset HF or HF decompensation during follow-up), and ischaemic stroke. In addition, a composite endpoint comprising all-cause mortality, heart failure, and ischaemic stroke was defined. Clinical events were obtained from the patients’ electronic medical records, including information from cardiology outpatient visits, primary care consultations, and emergency department attendances.

### 2.6. Statistical Analysis

Continuous variables are summarized as mean ± standard deviation (SD), or as median and interquartile range (IQR) when appropriate, following assessment of their distribution, while categorical variables are expressed as absolute frequencies and percentages. Comparisons across groups were performed using the chi-square test or Fisher’s exact test for categorical variables, as appropriate. For continuous variables, comparisons were performed using the non-parametric Kruskal–Wallis test.

Kaplan–Meier survival curves were generated, and comparisons between curves were performed using the log-rank test. Patients without the event of interest were censored at the end of follow-up or at the last available contact; for analyses in which mortality was not the event of interest, patients who died before experiencing the event were censored at the date of death. Univariable and multivariable Cox proportional hazards regression analyses were performed to identify factors associated with clinical outcomes during follow-up. Multivariable Cox models were used to assess the independent prognostic contribution of age and AF type for each clinical outcome after adjustment for relevant baseline clinical variables. Results are expressed as hazard ratios (HRs) with corresponding 95% confidence intervals (95% CIs).

Statistical significance was defined as a two-sided *p* value < 0.05.

## 3. Results

### 3.1. Baseline Characteristics

Among the 1001 patients enrolled in the REGUEIFA registry, four (0.4%) were excluded because of missing follow-up data. The final study population comprised 997 patients, stratified into four age groups: <75 years (*n* = 694, 69.6%), 75–84 years (*n* = 239, 23.4%), 85–89 years (*n* = 45, 4.5%), and ≥90 years (*n* = 19, 1.9%). Baseline characteristics are summarised in Table 1. Stratification by age revealed a clinical gradient associated with ageing.

Increasing age was associated with a higher proportion of women, a progressive increase in both thromboembolic and bleeding risk, reflected by significantly higher CHA_2_DS_2_-VASc and HAS-BLED scores, and a greater prevalence of comorbidities, including chronic kidney disease, anaemia, chronic obstructive pulmonary disease (COPD), cognitive impairment, valvular heart disease, and pulmonary hypertension.

Relevant age-related differences were also observed in AF characteristics and management. Paroxysmal and persistent AF were more common in younger patients, whereas long-standing persistent/permanent AF increased progressively with age, accounting for nearly 70% of cases among patients aged ≥90 years.

Similarly, treatment strategies changed substantially across age groups. Rhythm-control therapy decreased progressively from 79% in patients aged < 75 years to approximately 10% in those older than 85 years (*p* < 0.001). Interventions aimed at restoring or maintaining sinus rhythm, particularly electrical cardioversion and catheter ablation, were significantly more frequent in younger patients, whereas rate-control strategies and cardiac device implantation predominated in older individuals.

Although the overall use of oral anticoagulation was high across all age groups (overall prevalence > 85%), older patients were more frequently treated with vitamin K antagonists than with direct oral anticoagulants. No clinically relevant differences were observed in the main echocardiographic parameters or in anticoagulation control between age groups. Anticoagulation characteristics are shown in Table 2 and Table 3.

### 3.2. Clinical Outcomes

During follow-up, the incidence of adverse clinical outcomes increased progressively with age. Cardiovascular mortality rose from 3.5% in patients younger than 75 years to 26.3% among those aged ≥90 years (*p* < 0.001). Similarly, HF events increased from 11.7% to 42.1% (*p* < 0.001), whereas the incidence of the composite endpoint increased from 14.8% to 73.7% (*p* < 0.001). In contrast, the incidence of ischaemic stroke remained low and showed less marked differences across age groups (Table 4). During follow-up, 41 patients had undergone AF catheter ablation by the 1-year visit, increasing to 43 by the 2-year visit. At the corresponding follow-up visits, 32 (78.0%) and 36 (83.7%) of these patients, respectively, were in sinus rhythm.

**Figure 1:**
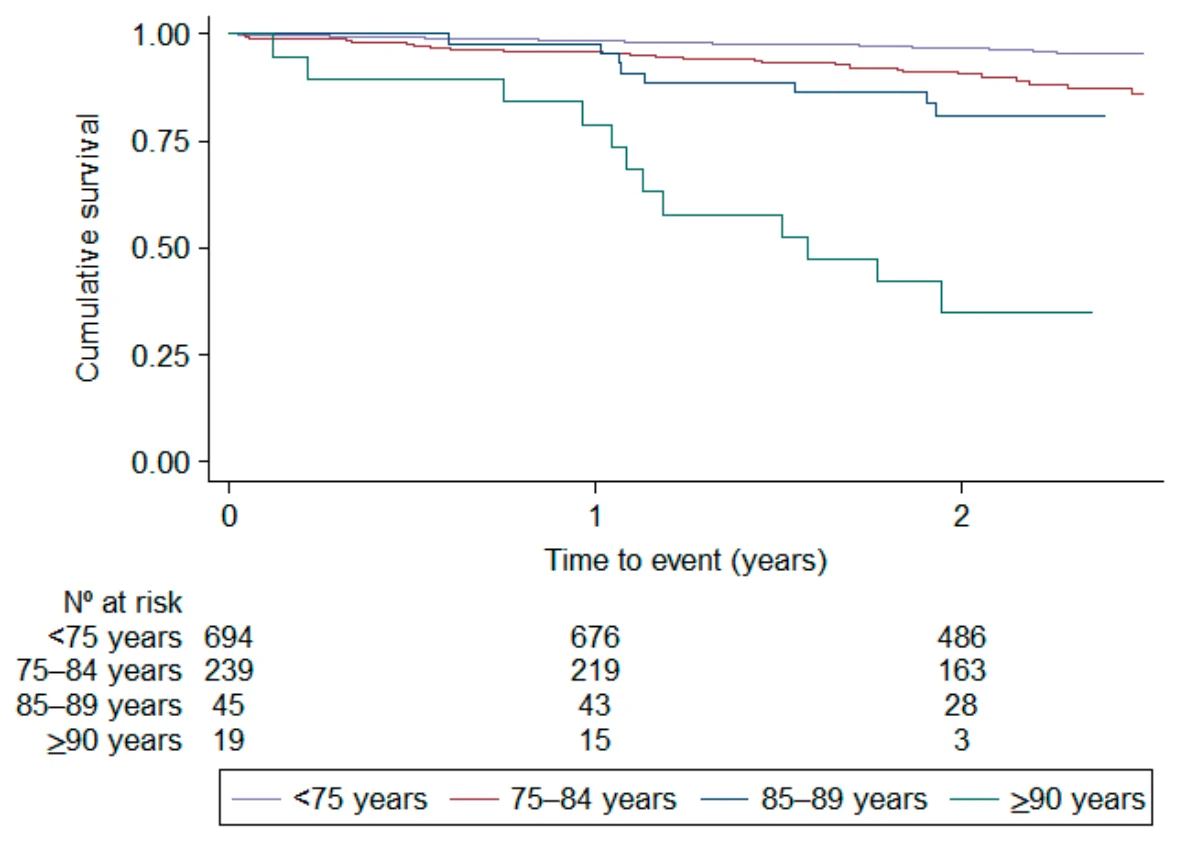
Kaplan–Meier curves for all-cause mortality according to age group. Log-rank *p* < 0.001.

**Figure 2:**
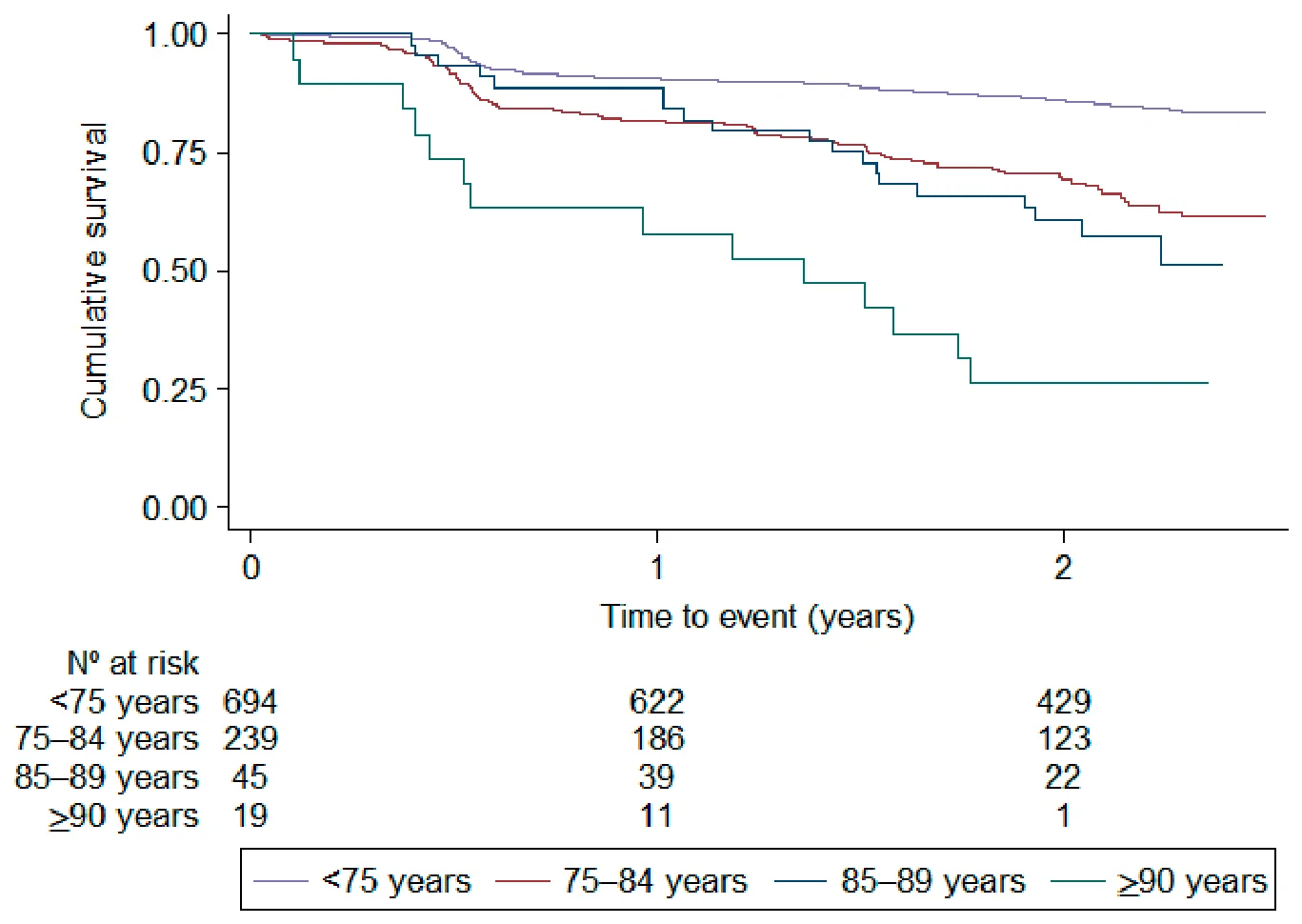
Kaplan–Meier curves for the composite endpoint according to age group. Log-rank *p* < 0.001.

Kaplan–Meier survival analyses confirmed this age-related prognostic gradient, demonstrating a progressive reduction in event-free survival with increasing age. Significant differences were observed for all-cause mortality and the composite endpoint (Figure 1 and Figure 2). Similarly, patients with long-standing persistent or permanent AF had a significantly higher incidence of all-cause mortality and the composite endpoint during follow-up than those with first-diagnosed, paroxysmal, or persistent AF.

Increasing age was consistently associated with a higher risk of adverse clinical outcomes in both unadjusted and adjusted analyses. In the unadjusted analyses, including Kaplan–Meier survival curves and incidence rate ratios, patients with long-standing persistent or permanent AF experienced a higher incidence of all-cause mortality and the composite endpoint than those with first-diagnosed, paroxysmal, or persistent AF. However, in the multivariable Cox regression models, AF type was no longer independently associated with any of the analysed outcomes.

In the final multivariable model for the composite endpoint (all-cause mortality, ischaemic stroke, or worsening HF), age was independently associated with an increased risk of events. Compared with patients younger than 75 years, those aged 75–84 years had approximately twice the risk (HR 2.01; 95% CI 1.47–2.76; *p* < 0.001), whereas patients aged 85–89 years had a 2.34-fold higher risk (95% CI 1.41–3.90; *p* = 0.001). The association was even stronger among patients aged ≥90 years, whose risk was 6.5-fold higher than that of the youngest group (HR 6.50; 95% CI 3.60–11.72; *p* < 0.001). Chronic kidney disease was also independently associated with the composite endpoint (HR 1.55; 95% CI 1.02–2.35; *p* = 0.042), whereas coronary artery disease showed a borderline association (HR 1.43; 95% CI 1.00–2.06; *p* = 0.052). No independent association was observed between AF type and the composite endpoint (Table 5A).

In an additional multivariable model incorporating treatment strategy (rhythm control vs. rate control), age remained independently associated with the composite endpoint (HR 1.04 per year; 95% CI 1.03–1.06; *p* < 0.001). Rhythm-control strategy was also independently associated with a lower risk of the composite endpoint (HR 0.50; 95% CI 0.34–0.74; *p* = 0.001) (Table 5B).

A similar pattern was observed for worsening HF. After multivariable adjustment, age remained independently associated with HF events, with an 84% higher risk among patients aged 75–84 years (HR 1.84; 95% CI 1.28–2.65; *p* = 0.001), an almost two-fold higher risk among those aged 85–89 years (HR 1.99; 95% CI 1.06–3.74; *p* = 0.031), and more than a four-fold higher risk among patients aged ≥90 years (HR 4.42; 95% CI 2.07–9.44; *p* < 0.001), compared with patients younger than 75 years. In addition, COPD or obstructive sleep apnoea syndrome was independently associated with worsening HF (HR 1.59; 95% CI 1.08–2.33; *p* = 0.019). AF type was not independently associated with HF events (Table 6A).

In an additional multivariable model incorporating treatment strategy (rhythm control vs. rate control), the risk of worsening HF increased by 3% for every additional year of age (HR 1.03; 95% CI 1.01–1.04; *p* = 0.003; Table 6B). Rhythm-control strategy was also independently associated with a lower risk of worsening HF (HR 0.47; 95% CI 0.30–0.74; *p* = 0.001) (Table 6B).

In the final multivariable model for all-cause mortality, age remained an independent predictor of death during follow-up (HR 1.09 per year; 95% CI 1.06–1.13; *p* < 0.001). Anaemia (HR 2.14; 95% CI 1.14–4.03; *p* = 0.018) and chronic kidney disease (HR 2.04; 95% CI 1.16–3.60; *p* = 0.013) were also independently associated with a higher risk of death. No significant differences in mortality were observed according to AF type after multivariable adjustment, whereas the risk of mortality increased by 9% for every additional year of age (Table 7A,B).

Additional multivariable Cox models including reduced left ventricular ejection fraction (LVEF ≤ 40%) were performed in the 932 patients with available LVEF data. Reduced LVEF was independently associated with the composite endpoint (HR 2.55; 95% CI 1.83–3.55; *p* < 0.001), worsening HF (HR 2.21; 95% CI 1.48–3.30; *p* < 0.001), and all-cause mortality (HR 2.95; 95% CI 1.70–5.12; *p* < 0.001), while the association between age and outcomes remained consistent (Table 8).

## 4. Discussion

In this multicentre real-world study of patients with AF, three main findings emerged. First, a clear age-related risk gradient was identified, characterised by a progressive worsening of the clinical profile and an increasing incidence of adverse events during follow-up. Second, although long-standing persistent or permanent AF was associated with a worse prognosis in unadjusted analyses, AF type lost its prognostic significance after adjustment for age and comorbidities, whereas age remained independently and consistently associated with mortality, worsening HF, and the composite endpoint. Third, rhythm-control strategy was independently associated with a lower risk of the clinical outcomes assessed.

Age stratification revealed a marked clinical and prognostic gradient. Increasing age was associated with a progressively greater burden of comorbidities, higher thromboembolic and bleeding risk, and a higher prevalence of sustained forms of AF, reflecting an increasingly complex clinical profile. This adverse baseline profile was accompanied by a stepwise increase in the incidence of mortality, worsening HF, and the composite endpoint during follow-up, consistent with a clear dose–response relationship between age and the risk of adverse clinical outcomes. This gradient was confirmed by survival analyses and, importantly, persisted after multivariable adjustment, with age emerging as an independent predictor of all analysed outcomes. Nonagenarian patients had a more than six-fold higher risk of the composite endpoint compared with patients younger than 75 years, whereas each additional year of age was associated with a 9% increase in the risk of mortality.

These findings are consistent with those reported in large contemporary registries such as EORP-AF and other recent observational studies, in which ageing has been associated with a greater burden of comorbidities, progressively higher thromboembolic and bleeding risk, and an increased incidence of adverse cardiovascular outcomes during follow-up [20,21]. From a pathophysiological perspective, this relationship probably reflects the interaction between ageing, progressive atrial remodelling, inflammation, fibrosis, and the accumulation of comorbidities. In this context, AF and ageing have been proposed to constitute a vicious cycle, each promoting the other and accelerating clinical deterioration [22]. Importantly, the inclusion of reduced LVEF (≤40%) in the multivariable models did not materially modify the association between advancing age and adverse outcomes, while reduced LVEF was independently associated with mortality, worsening HF, and the composite endpoint.

The prognostic significance of AF type remains a matter of debate. In our cohort, patients with non-paroxysmal AF experienced a higher incidence of adverse clinical outcomes in unadjusted analyses. However, this association disappeared after adjustment for age and other relevant clinical variables. These findings suggest that the excess risk observed in sustained forms of AF is largely determined by the clinical profile of the patients who develop them—characterised by older age and a greater burden of comorbidities—rather than by the temporal pattern of the arrhythmia itself.

These findings are also consistent with several contemporary registries. In the Loire Valley Atrial Fibrillation Project, the initial association between non-paroxysmal AF and a higher risk of stroke and mortality lost statistical significance after adjustment for age and comorbidities [7]. Similarly, the GARFIELD-AF registry showed that, among anticoagulated patients, the risks of stroke/systemic embolism and HF were comparable across AF patterns [23]. Likewise, landmark studies including the Euro Heart Survey, the SPORTIF analysis, and the Stockholm cohort had already demonstrated that the thromboembolic risk associated with paroxysmal AF is comparable to that of sustained forms when patients have a similar baseline risk profile [9,24,25]. More recently, Ntaios et al. found no differences in stroke risk between paroxysmal and non-paroxysmal AF among anticoagulated patients and even reported a higher incidence of myocardial infarction in patients with paroxysmal AF [26].

Nevertheless, not all studies have reported consistent findings. The Japanese Fushimi registry identified paroxysmal AF as an independent predictor of a lower incidence of stroke or systemic embolism compared with sustained forms [27]. In the GARFIELD-AF registry, non-paroxysmal AF patterns were associated with a greater risk of stroke, HF and death among patients who were not receiving anticoagulation [23]. However, differences between registries may be explained, at least in part, by methodological and clinical factors. First, the proportion of anticoagulated patients was substantially higher in our cohort (more than 85%) than in registries such as Fushimi and even GARFIELD-AF, a difference that likely attenuated prognostic disparities between AF patterns [23,27]. Second, the conventional classification into paroxysmal and non-paroxysmal AF represents a simplified approach to a disease that is inherently dynamic. Progression or regression of the arrhythmic pattern during follow-up, rhythm-control interventions, and, most importantly, the lack of information regarding the actual AF burden may all have substantially influenced the observed outcomes [28].

The absence of an independent prognostic effect of AF type reinforces the need for markers that more accurately reflect true exposure to the arrhythmia. In this regard, AF burden, defined as the total time spent in AF, may have greater prognostic relevance. Among patients with paroxysmal AF, Go et al. demonstrated that a higher AF burden was independently associated with an increased risk of ischaemic stroke [29]. Furthermore, considering AF as a dynamic disease may provide additional prognostic information. In the Fushimi registry, transition from sustained forms to paroxysmal AF was associated with a reduction in major cardiovascular events, highlighting the prognostic importance of temporal changes in AF pattern [30].

In parallel, the incorporation of biomarkers into clinical risk assessment has been proposed as a strategy to improve the predictive performance of current risk scores by providing a more accurate characterisation of thromboembolic risk beyond traditional clinical variables [31,32]. Likewise, the application of artificial intelligence and machine learning to ECG analysis and large clinical databases is enabling more accurate AF detection and improved individual risk stratification, with the potential to enhance early diagnosis and personalised patient management [33].

Treatment strategies differed across age groups, with less frequent use of rhythm-control strategies and procedures in older patients, where more advanced AF and a greater comorbidity burden may influence treatment selection. Rhythm-control strategy was independently associated with a lower risk of adverse clinical outcomes, consistent with the growing evidence supporting rhythm control in AF. Although current guidelines do not establish an upper age limit for catheter ablation, available evidence suggests higher rates of arrhythmia recurrence and procedure-related complications in older patients, supporting careful individualised selection rather than age-based exclusion of catheter ablation [34].

The findings of the present study have several important clinical implications. First, they support current guideline recommendations that prioritise comprehensive cardiovascular risk assessment over AF type when guiding therapeutic decisions. Second, they reinforce the central prognostic role of age, with a progressive effect extending beyond the conventional thresholds incorporated into current risk scores.

Finally, our findings suggest that strategies aimed at optimising the management of age-related comorbidities, particularly chronic kidney disease, anaemia, and HF, may have a greater impact on clinical outcomes than AF type itself, supporting an individualised approach to treatment selection and the potential value of rhythm-control strategies.

## 5. Conclusions

In this large prospective real-world cohort of patients with AF, advancing age was associated with a progressively worse clinical profile and a substantially higher risk of mortality, worsening HF, and the composite endpoint during follow-up. Although patients with long-standing persistent or permanent AF experienced poorer outcomes in unadjusted analyses, AF type was not independently associated with adverse clinical outcomes after adjustment for age and comorbidities. These findings suggest that age and the associated burden of comorbidities are more important determinants of prognosis than AF type, while rhythm-control strategy was independently associated with a lower risk of adverse clinical outcomes, reinforcing the need for risk stratification based on the patient’s overall clinical profile rather than AF type.

## 6. Limitations

This study has some limitations that should be acknowledged. First, its observational design precludes any inference of causality, and despite multivariable adjustment, residual confounding cannot be excluded. However, this reflects real-world clinical practice and allows the assessment of associations in an unselected population, enhancing the external validity of the findings. Second, AF type was classified at baseline and may change over time, and no systematic assessment of AF burden was available, limiting the ability to capture the dynamic nature of the arrhythmia. Nevertheless, this approach mirrors routine clinical practice, where AF classification is typically based on clinical presentation at the time of evaluation. Third, treatment strategies differed substantially across age groups, with less frequent use of rhythm-control strategies and rhythm-restoration procedures in older patients. This reflects real-world clinical practice and may limit the assessment of the independent effects of specific treatment strategies across age groups.

## Figures and Tables

**Table 1:** Baseline clinical characteristics stratified by age groups. AF, atrial fibrillation; AV, atrioventricular; BMI, body mass index; CHA_2_DS_2_-VASc, Congestive heart failure, Hypertension, Age, Diabetes, Stroke, Vascular disease, Age, Sex category score; COPD, chronic obstructive pulmonary disease; NYHA, New York Heart Association; OSA, obstructive sleep apnea; SD, standard deviation.

	Overall (*n* = 997)	<75 Years (*n* = 694)	75–84 Years (*n* = 239)	85–89 Years (*n* = 45)	≥90 Years (*n* = 19)	*p* Value
Patient characteristics						
Female sex, *n* (%)	321 (32.20)	191 (27.52)	106 (44.35)	15 (33.33)	9 (47.37)	<0.001
BMI (kg/m^2^), median (IQR)	28.94 (26.23–32.30)	29.36 (26.57–32.77)	28.37 (25.81–31.22)	28.07 (25.39–30.61)	24.86 (23.74–29.78)	<0.001
Cardiovascular risk factors						
Current smoker, *n* (%)	86 (8.63)	79 (11.38)	7 (2.93)	0 (0.00)	0 (0.00)	<0.001
Alcohol consumption, *n* (%)	584 (58.58)	436 (62.82)	115 (48.12)	23 (51.11)	10 (52.63)	0.001
Hypertension, *n* (%)	622 (62.39)	388 (55.91)	183 (76.57)	35 (77.78)	16 (84.21)	<0.001
Diabetes mellitus, *n* (%)	189 (18.96)	117 (16.86)	57 (23.85)	11 (24.44)	4 (21.05)	0.073
Dyslipidemia, *n* (%)	481 (48.24)	320 (46.11)	134 (56.07)	21 (46.67)	6 (31.58)	0.026
Obesity (BMI ≥ 30 kg/m^2^), n (%)	408 (40.92)	302 (43.52)	87 (36.40)	15 (33.33)	4 (21.05)	0.045
Thromboembolic and bleeding risk						
CHA_2_DS_2_-VASc score, median (IQR)	2 (1–3)	2 (1–3)	4 (3–4)	3 (3–4)	4 (3–4)	<0.001
HAS-BLED score, median (IQR)	1 (0–1)	0 (0–1)	1 (1–2)	1 (1–2)	2 (1–2)	<0.001
Comorbidities						
COPD, *n* (%)	107 (10.73)	48 (6.92)	47 (19.67)	7 (15.56)	5 (26.32)	<0.001
Obstructive sleep apnea, *n* (%)	50 (5.02)	43 (6.20)	4 (1.67)	1 (2.22)	2 (10.53)	0.010
COPD or OSA, *n* (%)	145 (14.54)	80 (11.53)	51 (21.34)	8 (17.78)	6 (31.58)	<0.001
Dementia, *n* (%)	7 (0.70)	0 (0.00)	3 (1.26)	2 (4.44)	2 (10.53)	<0.001
Malignancy, *n* (%)	83 (8.32)	35 (5.04)	34 (14.23)	13 (28.89)	1 (5.26)	<0.001
Anemia, *n* (%)	40 (4.01)	17 (2.45)	14 (5.86)	7 (15.56)	2 (10.53)	<0.001
Chronic kidney disease, *n* (%)	64 (6.42)	26 (3.75)	28 (11.72)	6 (13.33)	4 (21.05)	<0.001
Cardiovascular history						
Previous heart failure, *n* (%)	148 (14.84)	95 (13.69)	43 (17.99)	8 (17.78)	2 (10.53)	0.365
NYHA functional class ≥II†, *n* (%)	121/148 (81.76)	72/95 (75.79)	39/43 (90.70)	8/8 (100.00)	2/2 (100.00)	0.097
Coronary artery disease, *n* (%)	115 (11.53)	73 (10.52)	35 (14.64)	7 (15.56)	0 (0.00)	0.096
Valvular heart disease, *n* (%)	114 (11.43)	45 (6.48)	55 (23.01)	10 (22.22)	4 (21.05)	<0.001
Cardiomyopathy, *n* (%)	78 (7.82)	47 (6.77)	29 (12.13)	2 (4.44)	0 (0.00)	0.036
Congenital heart disease, *n* (%)	4 (0.40)	3 (0.43)	1 (0.42)	0 (0.00)	0 (0.00)	1.000
Pulmonary hypertension, *n* (%)	30 (3.01)	13 (1.87)	12 (5.02)	4 (8.89)	1 (5.26)	0.006
Previous thromboembolic and bleeding events						
Previous thromboembolic events, *n* (%)	61 (6.12)	36 (5.19)	20 (8.37)	3 (6.67)	2 (10.53)	0.182
Previous ischemic stroke, *n* (%)	29 (2.91)	16 (2.31)	12 (5.02)	0 (0.00)	1 (5.26)	0.086
Previous bleeding events, *n* (%)	35 (3.51)	17 (2.45)	11 (4.6)	4 (8.89)	3 (15.79)	0.003
Laboratory findings						
Serum creatinine (mg/dL), median (IQR)	0.91 (0.79–1.09)	0.90 (0.78–1.06)	0.93 (0.80–1.15)	1.03 (0.83–1.31)	1.04 (0.90–1.30)	<0.001
AF characteristics						
Clinical AF type						<0.05
First-diagnosed AF	227 (22.77)	158 (22.77)	55 (23.01)	11 (24.44)	3 (15.79)	
Paroxysmal AF	223 (22.37)	174 (25.07)	41 (17.15)	5 (11.11)	3 (15.79)	
Persistent AF	263 (26.38)	238 (34.29)	22 (9.21)	3 (6.67)	0 (0.00)	
Long-standing persistent/permanent AF	284 (28.49)	124 (17.87)	121 (50.63)	26 (57.78)	13 (68.42)	
Treatment strategy						<0.001
Rhythm-control strategy	632 (63.39)	548 (78.96)	77 (32.22)	5 (11.11)	2 (10.53)	
Rate-control strategy	365 (36.61)	146 (21.04)	162 (67.78)	40 (88.89)	17 (89.47)	
Planned or performed rhythm restoration procedures	545 (54.66)	474 (68.30)	61 (25.52)	5 (11.11)	5 (26.32)	<0.001
Electrical cardioversion	241 (24.17)	213 (30.69)	26 (10.88)	2 (4.44)	0 (0.00)	<0.001
Pharmacological cardioversion	26 (2.61)	19 (2.74)	7 (2.93)	0 (0.00)	0 (0.00)	0.854
Catheter ablation	167 (16.75)	161 (23.20)	6 (2.51)	0 (0.00)	0 (0.00)	<0.001
Cardiac device implantation	39 (3.91)	10 (1.44)	20 (8.37)	4 (8.89)	5 (26.32)	<0.001
AV node ablation	9 (0.90)	5 (0.72)	3 (1.26)	1 (2.22)	0 (0.00)	0.381

**Table 2:** Anticoagulation therapy according to age group. DOAC, direct oral anticoagulant; VKA, vitamin K antagonist.

	<75 Years (*n* = 694)	75–84 Years (*n* = 239)	85–89 Years (*n* = 45)	≥ 90 Years (*n* = 19)	*p* Value
Receiving oral anticoagulation, n (%)	609 (87.75)	232 (97.07)	41 (91.11)	16 (84.21)	<0.001
Type of oral anticoagulant					<0.001
VKA	308/609 (50.57)	173/232 (74.57)	34/41 (82.93)	12/16 (75.00)	
DOAC	301/609 (49.43)	59/232 (25.43)	7/41 (17.07)	4/16 (25.00)	
Type of DOAC					0.017
Dabigatran	112/301 (37.21)	15/59 (25.42)	1/7 (14.29)	1/4 (25.00)	
Edoxaban	34/301 (11.30)	14/59 (23.73)	0/7 (0.00)	1/4 (25.00)	
Rivaroxaban	90/301 (29.90)	11/59 (18.64)	2/7 (28.57)	1/4 (25.00)	
Apixaban	65/301 (21.59)	19/59 (32.20)	4/7 (57.14)	1/4 (25.00)	

**Table 3:** Quality of anticoagulation control with vitamin K antagonists according to age group. INR, international normalized ratio; SD, standard deviation; TTR, time in therapeutic range; VKA, vitamin K antagonist.

	<75 Years (*n* = 694)	75–84 Years (*n* = 239)	85–89 Years (*n* = 45)	≥ 90 Years (*n* = 19)	*p* Value
Receiving VKA therapy, n (%)	274/688 (39.83)	149/238 (62.61)	28/45 (62.22)	11/19 (57.89)	<0.001
Mean INR (last six measurements)					0.818
*n*	231	126	23	8	
Mean ± SD	2.52 ± 0.48	2.56 ± 0.51	2.49 ± 0.49	2.42 ± 0.27	
Time in therapeutic range (TTR) estimated using the Rosendaal method					0.649
*n*	256	137	25	10	
Mean ± SD	54.97 ± 26,91	54.17 ± 25.28	58.27 ± 25.91	45.59 ± 27.08	
TTR > 60%, n (%)	111/256 (43.36)	57/137 (41.61)	12/25 (48.00)	4/10 (40.00)	0.937

**Table 4:** **Clinical outcomes during follow-up stratified by age group.** The composite endpoint was defined as the occurrence of all-cause mortality, ischemic stroke, or worsening heart failure.

	Overall (*n* = 997)	<75 Years (*n* = 694)	75–84 Years (*n* = 239)	85–89 Years (*n* = 45)	≥90 Years (*n* = 19)	*p* Value
All-cause mortality, *n* (%)	76 (7.62)	27 (3.89)	29 (12.13)	8 (17.78)	12 (63.16)	<0.001
Cardiovascular mortality, *n* (%)	35 (3.51)	14 (2.02)	14 (5.86)	2 (4.44)	5 (26.32)	<0.001
Ischemic stroke, *n* (%)	13 (1.30)	5 (0.72)	8 (3.35)	0 (0.00)	0 (0.00)	0.034
Worsening heart failure, *n* (%)	159 (15.95)	81 (11.67)	58 (24.27)	12 (26.67)	8 (42.11)	<0.001
Composite endpoint, *n* (%)	219 (21.97)	103 (14.84)	83 (34.73)	19 (42.22)	14 (73.68)	<0.001

**Table 5:** Multivariable Cox regression model for the composite endpoint. HR, Hazard ratio; CI, confidence interval; AF, Atrial fibrillation.

(**A**) **Model with age stratified.**				
	HR	95% CI		*p* value
Age groups				
<75 years	Reference			
75–84 years	2.01	1.47	2.76	<0.001
85–89 years	2.34	1.41	3.90	0.001
≥90 years	6.50	3.60	11.72	<0.001
Type of AF				
First diagnosis	Reference			
Paroxysmal	0.70	0.46	1.08	0.108
Persistent	0.69	0.44	1.08	0.107
Long-standing persistent + Permanent	1.11	0.78	1.58	0.549
Chronic kidney disease	1.55	1.02	2.35	0.042
Coronary artery disease	1.43	1.00	2.06	0.052
(**B**) **Additional model including treatment strategy, with age as a continuous variable.**				
	HR	95% CI		*p* value
Age (years)	1.04	1.03	1.06	<0.001
Type of AF				
First diagnosis	Reference			
Paroxysmal	0.99	0.63	1.56	0.980
Persistent	0.93	0.58	1.49	0.764
Long-standing persistent + Permanent	0.95	0.66	1.37	0.786
Treatment strategy: Rhythm control	0.50	0.34	0.74	0.001

**Table 6:** Multivariable Cox regression model for worsening heart failure. HR, Hazard ratio; CI, confidence interval; AF, Atrial fibrillation; COPD, Chronic obstructive pulmonary disease; OSA, Obstructive sleep apnea.

(**A**) **Model with age stratified.**				
	HR	95% CI		*p* value
Age groups				
<75 years	Reference			
75–84 years	1.84	1.28	2.65	0.001
85–89 years	1.99	1.06	3.74	0.031
≥90 years	4.42	2.07	9.44	<0.001
Type of AF				
First diagnosis	Ref.			
Paroxysmal	0.62	0.38	1.02	0.060
Persistent	0.65	0.39	1.07	0.090
Long-standing persistent + Permanent	0.94	0.63	1.40	0.744
COPD or OSA	1.59	1.08	2.33	0.019
(**B**) **Additional model including treatment strategy, with age as a continuous variable.**				
	HR	95% CI		*p* value
Age (years)	1.03	1.01	1.04	0.003
Type of AF				
First diagnosis	Reference			
Paroxysmal	0.83	0.50	1.39	0.485
Persistent	0.82	0.48	1.38	0.449
Long-standing persistent + Permanent	0.76	0.50	1.15	0.196
COPD or OSA	1.49	1.02	2.19	0.040
Treatment strategy: Rhythm control	0.47	0.30	0.74	0.001

**Table 7:** Multivariable Cox regression model for all-cause mortality. HR, Hazard ratio; CI, confidence interval; AF, Atrial fibrillation.

(**A**) **Model with age stratified.**				
	HR	95% CI		*p* value
Age groups				
<75 years	Reference			
75–84 years	2.14	1.21	3.78	0.009
85–89 years	2.52	1.07	5.93	0.034
≥90 years	16.83	7.80	36.28	<0.001
Type of AF				
First diagnosis	Reference			
Paroxysmal	0.91	0.40	2,04	0.813
Persistent	0.78	0.32	1.93	0.595
Long-standing persistent + Permanent	1.38	0.71	2.68	0.346
Anemia	2.04	1.04	4.00	0.038
Chronic kidney disease	2.28	1.28	4.06	0.005
Coronary artery disease	1.84	1.02	3.33	0.043
Pulmonary hypertension	2.27	1.12	4.62	0.023
(**B**) **Model with age as a continuous variable.**				
	HR	95% CI		*p* value
Age (years)	1.09	1.06	1.13	<0.001
Type of AF				
First diagnosis	Reference			
Paroxysmal	1.21	0.54	2.71	0.649
Persistent	1.20	0.48	2.98	0.694
Long-standing persistent + Permanent	1.62	0.86	3.05	0.132
Anemia	2.14	1.14	4.03	0.018
Chronic kidney disease	2.04	1.16	3.60	0.013

**Table 8:** Multivariable Cox regression models including reduced LVEF. HR, Hazard ratio; CI, confidence interval; AF, Atrial fibrillation; LVEF, left ventricular ejection fraction.

(**A**) **Composite endpoint (all-cause mortality, ischaemic stroke, or worsening HF)**				
	HR	95% CI		*p* value
Age (years)	1.04	1.03	1.06	<0.001
Type of AF				
First diagnosis	Reference			
Paroxysmal	1.16	0.72	1.86	0.537
Persistent	1.04	0.64	1.67	0.881
Long-standing persistent + Permanent	0.94	0.65	1.38	0.769
Treatment strategy: Rhythm control	0.52	0.35	0.77	0.001
Reduced LVEF (LVEF ≤40%)	2.55	1.83	3.55	<0.001
(**B**) **Worsening HF**				
	HR	95% CI		*p* value
Age (years)	1.03	1.01	1.04	0.002
Type of AF				
First diagnosis	Reference			
Paroxysmal	0.97	0.57	1.67	0.915
Persistent	0.92	0.54	1.58	0.772
Long-standing persistent + Permanent	0.81	0.52	1.25	0.338
Treatment strategy: Rhythm control	0.49	0.31	0.79	0.003
Reduced LVEF (LVEF ≤40%)	2.21	1.48	3.30	<0.001
(**C**) **All-cause mortality**				
	HR	95% CI		*p* value
Age (years)	1.10	1.07	1.14	<0.001
Type of AF				
First diagnosis	Reference			
Paroxysmal	1.09	0.46	2.57	0.842
Persistent	1.16	0.47	2.86	0.748
Long-standing persistent + Permanent	1.47	0.78	2.79	0.236
Chronic kidney disease	2.14	1.19	3.88	0.012
Reduced LVEF (LVEF ≤40%)	2.95	1.70	5.12	<0.001

## Data Availability

Data will be made available on reasonable request to the corresponding author.
